# The mechanism of *Laceyella sacchari* FBKL4.010 produced tetramethylpyrazine in the liquid fermentation by comparative transcriptomic techniques

**DOI:** 10.3389/fmicb.2024.1414203

**Published:** 2024-06-13

**Authors:** Xiaodan Wang, Wu Huang, Jin Huang, Xiaoye Luo, Mingbo Nie, Tao Jiang, Shidong Ban, Pei Li

**Affiliations:** ^1^School of Liquor and Food Engineering, Guizhou University, Guiyang, China; ^2^Guizhou Provincial Key Laboratory of Fermentation Engineering and Biological Pharmacy, Guizhou University, Guiyang, China; ^3^Guizhou Anjiu Co., Ltd., Zunyi, China; ^4^Academy of Agricultural Planning and Engineering, Beijing, China; ^5^Qiandongnan Engineering and Technology Research Center for Comprehensive Utilization of National Medicine, Kaili University, Kaili, China

**Keywords:** Moutai-flavor liquor, tetramethylpyrazine, *Laceyeella sacchari* FBKL4.010, liquid fermentation, comparative transcriptomic

## Abstract

Tetramethylpyrazine (TTMP) is considered a crucial flavor component in Moutai-flavored liquor. *Laceyeella sacchari* FBKL4.010 (*L. sacchari*) is the dominant species found in Moutai-flavor Daqu, and this study aims to determine the mechanism by which *L. sacchari* produces TTMP during liquid fermentation of Moutai-flavor Daqu. The results of the liquid fermentation performance demonstrated a gradual increase in biomass over time, while there was a gradual decline in residual glucose content and pH value. Furthermore, analysis of volatile components revealed that liquid fermentation significantly enhanced the production of TTMP in Moutai-flavor Daqu, with the relative content of TTMP reaching 14.24 mg/L after 96 h of liquid fermentation. Additionally, to explore the synthesis mechanism of TTMP, we compared differentially expressed genes (DEGs) of *L. sacchari* between 24 and 96 h using comparative transcriptomic techniques. The results indicated that DEGs involved in isoleucine, valine, and leucine biosynthesis pathway were upregulated, while those associated with isoleucine, valine, and leucine degradation pathway were downregulated, suggesting that the valine, leucine, and isoleucine biosynthesis pathway primarily contributes ammonia for TTMP synthesis. The findings of this study present an opportunity for further elucidating the production process of TTMP in Moutai-flavor Daqu during liquid fermentation.

## 1 Introduction

Chinese liquor, known as baijiu in China, which is globally recognized as one of the top six distilled alcoholic beverages (alongside Brandy, Whisky, Vodka, Gin and Rum), holds a prominent position in traditional fermented foods and continues to be favored by modern individuals despite its thousands of years of heritage (Xu et al., 2017; Shi et al., 2022). This not only showcases the allure and value of Chinese liquor but also highlights its significance as a health product (Zheng and Han, 2016). Daqu, serving as the carrier of microbial fermentation, plays a pivotal role in Chinese liquor production by providing a wide range of microbes and numerous enzymes for the fermentation process, significantly impacting the flavor, quality, and functionality of Chinese liquor (Zuo et al., 2018; Liu et al., 2023). The microbial composition of Daqu mainly comprises bacteria, molds, and yeasts (He et al., 2019; Cai et al., 2021). The presence of specific functional microbes and the implementation of a unique brewing process can significantly enhance the contents of flavor substances, including phenols, terpenes, pyrazines, amino acids, and polypeptides, throughout the production of Chinese liquor (Shi et al., 2022). Therefore, exploring microbial fermentation products in Daqu is significant for improving the fermentation quality and flavor characteristics of Chinese liquor.

Tetramethylpyrazine (TTMP), a pyrazine substance, is regarded as a crucial flavor component within Chinese liquor (Fan et al., 2007; Shi et al., 2022). It is widely present in raw foods, processed foods, and alcoholic beverages alike (Shi et al., 2022; Tong et al., 2023). Possessing an enjoyable aroma reminiscent of roasted peanuts, hazelnuts, and cocoa beans makes TTMP an essential aromatic compound (Liu et al., 2024; Zhang, W. Q., et al., 2019). Apart from serving as a flavor enhancer in food products, TTMP holds significant nutritional value as well (Shi et al., 2022). Henceforth, TTMP not only contributes significantly to the flavor profile of Chinese liquor but also imparts it with various health benefits (Hao et al., 2013; Cui et al., 2020). Solid-state fermentation experiment showed that TTMP, an important flavor component of Moutai-flavor liquor, accounted for 52.68% of the volatile flavor components, confirming the ability of TTMP production under solid-state fermentation (Li et al., 2019). The biosynthesis of TTMP, which is produced through the thermodynamic reaction involving ammonia deaminized from amino acids by dehydrogenase and acetoin synthesized by both α-acetolactate synthase and α-acetolactate decarboxylase catalyzed pyruvic acid converted by glucose through EMP pathway, has been primarily studied in Bacillus species such as *Bacillus subtilis*, *Bacillus licheniformis*, and *Bacillus amyloliquefaciens* (Larroche et al., 1999; Sun et al., 2023). The biosynthesis performance of TTMP in liquid fermentation is generally superior to that in solid fermentation due to the advantages offered by the former, including homogeneity and enhanced control over the fermentation process and feeding. Therefore, liquid fermentation technology is widely used in Chinese liquor industry (He et al., 2019).

*Thermoactinomycetes* is a kind of thermophilic, aerobic, and chemotrophic gram-positive bacterium, which usually exists in high temperature environments such as terrestrial thermal springs, high temperature Daqu and compost (Matsuo et al., 2006). In recent years, due to the in-depth study of high-temperature Daqu by Chinese scholars, there have been many reports about thermophilic microorganisms (Yao et al., 2014; Li et al., 2019). Meanwhile, previous researches have demonstrated that TTMP may be generated through microbial metabolism during Chinese liquor fermentation (Meng et al., 2016; Xu et al., 2018). In our previous study, we reported a *Thermoactinomycetes* strain of *Laceyeella sacchari* FBKL4.010 (*L. sacchari*) screened from Moutai-flavor Daqu, which has the ability to produce TTMP (Li et al., 2018). However, the mechanism of *L. sacchari* produces TTMP in liquid fermentation remains unclear.

The present study focuses on a preliminary investigation into the liquid-based fermentation production of TTMP using *L. sacchari*, with emphasis on the fermentation properties and volatile components during the process. Additionally, we have employed the comparative transcriptomic technology to analyze its metabolic pathways.

## 2 Materials and methods

### 2.1 Strains and culture conditions

*L. sacchari* was cultivated at 45°C and continuously shaken at 160 rpm in ISP2 medium (4 g/L yeast extract, 10 g/L malt extract, and 4 g/L glucose, pH 7.0). Then, the cultivated *L. sacchari* was transferred to the liquid fermentation medium (100 g/L glucose, 30 g/L peptone, 5 g/L NaCl, 4 g/L K_2_HPO_3_, 2 g/L KH_2_PO_3_, and 0.5 g/L MgSO_4_·7H_2_O, pH 7.0) and continuously fermented at 45°C and 160 rpm for 24, 48, 72, 96, and 120 h, respectively.

### 2.2 Determination of the fermentation liquid performance

The fermentation liquid was accurately collected and subjected to filtration for the removal of the supernatant. The resulting filtrate was utilized for determination of residual sugar content, pH value, and biomass following established protocols (Wang et al., 2012).

### 2.3 Determination of the volatile components

The samples were placed in a headspace bottle containing 3 g of sodium chloride and 1 μL of internal standard 2-octanol (0.822 mg/mL) to maintain a temperature of 60°C for 15 min, facilitating gas chromatography-mass spectrometer (GC-MS) analysis. The GC-MS analysis was conducted using an Agilent Technologies gas chromatograph model 7890A equipped with a model 5957C mass selective detector and a model 7683B auto sampler (Agilent Technologies, Palo Alto, CA, United States). The injection temperature was set at 200°C. A DB-WAX column (30 m × 250 μm × 0.25 μm) was employed, and helium gas flowed constantly at a rate of 1 mL/min. The oven temperature was initially held at 30°C for a duration of 2 min, then increased to 50°C at a rate of 2°C/min and maintained for another 1 min, finally ramped up to the final temperature of 200°C at a rate of 5°C/min and kept constant for an additional period of 5 min. The ion source temperature was set to be maintained at precisely 230 °C throughout the analysis process. A mass range from *m*/*z* 20–550 and a detector voltage of 1.766 kV were utilized. The volatile constituents were identified by comparison with the standard mass spectrometry database from the NIST2011 database libraries (Xu et al., 2023).

### 2.4 RNA extraction and Illumina sequencing

After 24 and 96 h of fermentation, the total RNA of *L. sacchari* was extracted using the TRIzol-based method (Life Technologies, Grand Island, NY, United States). The quality control of RNA was conducted through several steps: (1) Monitoring the degree of RNA degradation and potential contamination on 1% agarose gels; (2) Checking RNA purity (OD_260_/OD_280_ and OD_260_/OD_230_) using the NanoPhotometer spectrophotometer (IMPLEN, CA, United States); (3) Assessing RNA integrity with Bioanalyzer 2100 (Agilent, Santa Clara, CA, United States).

The rRNA was depleted from 1 mg of total RNA using the Illumina MRZB12424 Ribo-Zero rRNA Removal Kit for bacteria (Illumina, San Diego, CA, United States). Then, the first-strand cDNA was synthesized using the ProtoScript II Reverse Transcriptase (New England BioLabs, Ipswich, MA, United States) at 25°C for 10 min, 42°C for 15 min, and 70°C for 15 min. The second-strand cDNA was synthesized using the NEBNext Second Strand Synthesis Reaction Buffer and dATP, dGTP, dCTP, dUTP mix (New England BioLabs, Ipswich, MA, United States) at 16°C for 1 h. Resulted cDNA was purified with Agencourt AMPure XP beads (Beckman Coulter, Brea, CA) and end repaired with NEBNext End Repair Reaction Buffer and Enzyme Mix (New England BioLabs, Ipswich, MA, United States) at 20°C for 30 min and 65°C for 30 min. Sequencing adapters were ligated using NEBNext Adaptor for Illumina (New England BioLabs, Ipswich, MA, United States) at 20°C for 15 min. The second-strand cDNA was then degraded using the USER enzyme mix (New England BioLabs, Ipswich, MA, United States) at 37°C for 15 min and the product was purified by Agencourt AMPure XP beads (Beckman Coulter, Brea, CA, United States). Finally, the clustering of the index-coded samples was performed on a cBot Cluster Generation System using NEBNext Q5 Hot Start HiFi PCR Master Mix (New England Biolabs, Ipswich, MA, United States). After cluster generation, sequencing was performed using the Illumina Novaseq 6000 platform (Modi et al., 2021). The raw data was deposit at National Center for Biotechnology Information (NCBI, https://www.ncbi.nlm.nih.gov/) with BioProject ID: PRJNA1048649.

### 2.5 Analysis of differentially expressed genes

Raw data were filtered by removing reads with ≥10% unidentified nucleotides (N), > 50% bases having PHRED quality scores of ≤20, and aligned to the barcode adapter using FASTP (version 0.18.0) (Chen et al., 2018). Quality trimmed reads were mapped to the reference genome (GenBank accession number: CP025943.1) using Bowtie2 (version 2.2.8) (Langmead and Salzberg, 2012) allowing no mismatches, reads mapped to ribosome RNA were removed. Retained reads were aligned with the genome using Bowtie2 (version 2.2.8) to identify known genes and calculated gene expression by RSEM (Li and Dewey, 2011).

The gene expression level was further normalized by using the fragments per kilobase of transcript per million (FPKM) mapped reads method to eliminate the influence of different gene lengths and amount of sequencing data on the calculation of gene expression (Mortazavi et al., 2008). The edgeR package^1^ was used to identify differentially expressed genes (DEGs) across samples with fold changes ≥2 and a false discovery rate adjusted *P* (*q* value) < 0.05 (Wang et al., 2021).

### 2.6 Bioinformatics analysis of DEGs

Gene Ontology (GO) annotation, which includes biological processes (BP), cellular components (CC), and molecular function (MF), as well as the Kyoto Encyclopedia of Genes and Genomes (KEGG) pathway enrichment analysis for the DEGs, were performed using http://www.geneontology.org/ and http://www.genome.jp/Pathway, respectively (Yu et al., 2018; Zhang et al., 2023).

### 2.7 Statistical analysis

The statistical significance was analyzed using Graphpad Prism 9.0 software (San Diego, CA, United States) with one-way analysis variance, and the data were expressed as the mean ± standard deviations (SD). All experiments were performed in triplicate.

## 3 Results and discussion

### 3.1 Liquid fermentation performance

**Figure 1:**
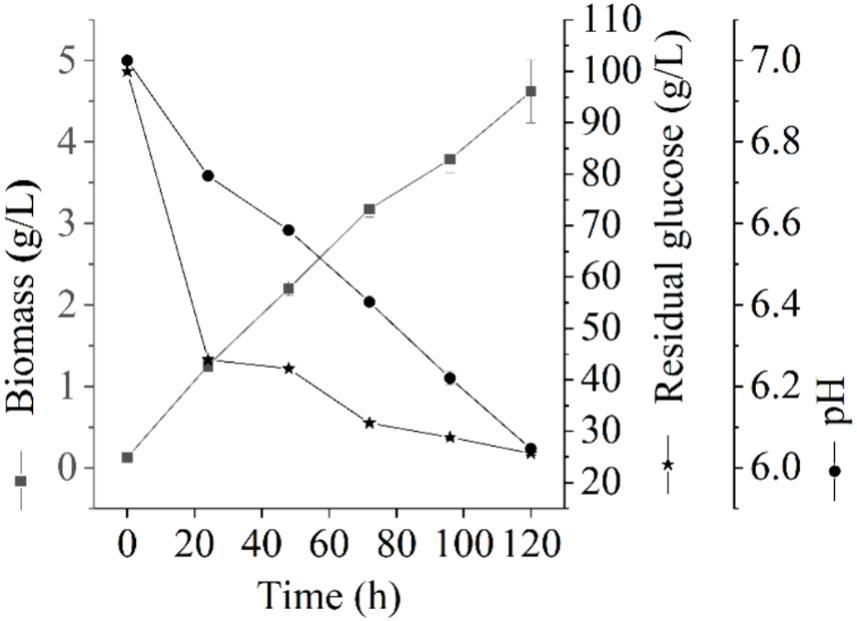
Changes of physicochemical properties at different fermentation stages.

The growth of *Thermoactinomycetes* generally had a long delay period in the early culture process, which greatly prolonged the fermentation period (Li et al., 2015). In this study, we explore the fermentation performance of *L. sacchari* in the liquid fermentation process and the results of the residual glucose content, pH value, and biomass of samples at different fermentation stages are shown in Figure 1. As shown in Figure 1, the biomass of *L. sacchari* gradually increased with the fermentation time, which was probably related to thickening of cell walls and membranes after the strain received acid stress (Shabala and Ross, 2008; Li et al., 2015). Glucose is the major carbon source for cellular biosynthesis and energy generation for many bacteria to the synthesis of serine, alanine, glycerol phosphate, etc. (Mulukutla et al., 2016). Figure 1 illustrates the rapid initial glucose consumption during the first 24 h of liquid fermentation. The glucose content decreased from an initial concentration of 100 g/L to 43.89 g/L, with a utilization rate of 56.11%. Subsequently, there was a gradual decrease in glucose consumption, reaching a concentration of 25.70 g/L at 120 h of fermentation with a utilization rate of 74.3%. These findings demonstrate that the majority of carbon sources were utilized for cell growth and metabolism. This trend is reflected in the biomass changes observed during the first 72 h, where it rapidly increased to reach a concentration of 3.18 g/L and further increased to reach 4.62 g/L at the end of the fermentation period. Moreover, pH value serves as a crucial parameter for assessing microbial growth and metabolism in a specific milieu (Husson, 2013). As depicted in Figure 1, the pH of *L. sacchari* during liquid fermentation exhibited a gradual decline from an initial value of 7.0 to 6.41 at 72 h and further to 6.05 at 120 h, indicating the production of organic acids by the strain during fermentation.

### 3.2 Volatile components analysis during the fermentation process

**Figure 2:**
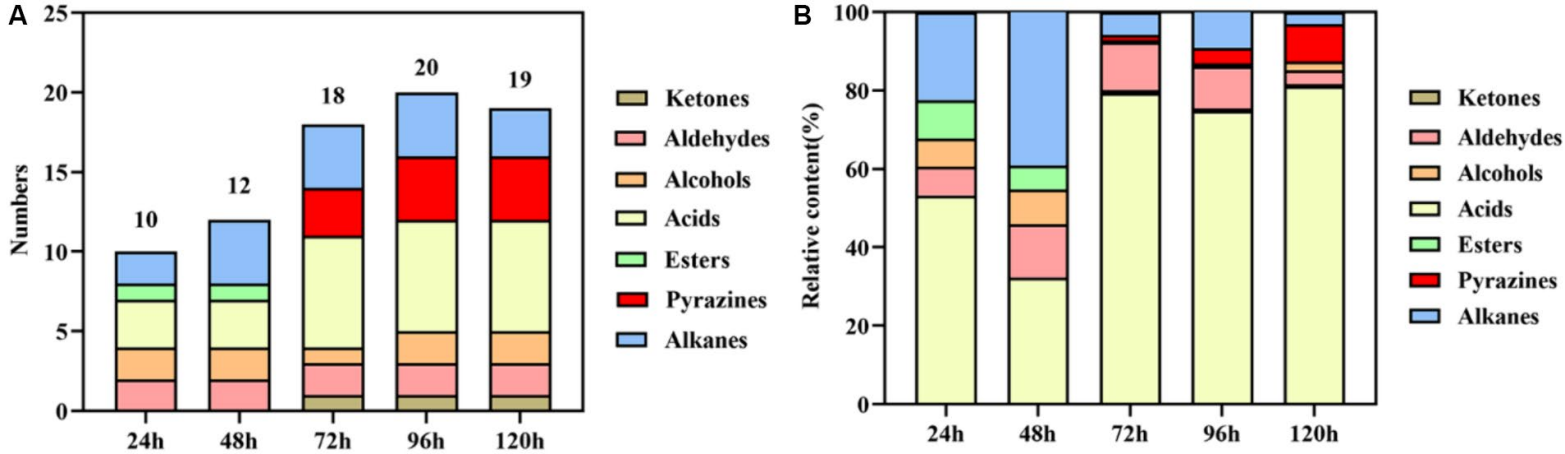
Statistical analysis of the numbers **(A)** and relative content **(B)** of volatile components during the fermentation process.

The results of volatile components at different fermentation stages are presented in Table 1 and Figure 2. Table 1 demonstrates the detection of a total of 27 volatile components, including ketones, aldehydes, alcohols, acids, esters, pyrazines, and alkanes during the fermentation process. Some of these components are also significant flavor substances in Chinese traditional liquors (Wei et al., 2020). In terms of fermentation duration, the early and middle stages predominantly consist of aldehydes, alcohols, esters, and alkanes, while the late stage is characterized by ketones, acids, and pyrazines. Meanwhile, from the perspective of the number of volatile components (Figure 2A), the number of volatile components reached the maximum at 96 h of fermentation. The analysis of relative contents (Figure 2B) reveals that during the fermentation process at both 24 and 48 h, the major volatile components include aldehydes, alcohols, acids, esters, and alkanes. However, from 72 to 120 h into the fermentation process, acid becomes the predominant volatile component with a proportion exceeding 70%. The relative contents of pyrazines gradually increase to 1.46, 3.95, and 9.46% at 72, 96, and 120 h, respectively. It is noteworthy that the relative content of TTMP reaches as high as 14.24 mg/L after a liquid fermentation period of 96 h.

**Table 1:** The volatile component contents of fermentation broth detected by GC-MS during the fermentation process (mean ± SD).

Category	Volatile components	Content (mg/L)				
		24 h	48 h	72 h	96 h	120 h
Ketones	Acetoin	—	—	38.22 ± 0.15	20.49 ± 4.04	10.31 ± 0.58
Aldehydes	1-Nonanal	3.41 ± 0.15	—	—	—	—
	Furfural	2.32 ± 0.38	53.01 ± 2.88	71.52 ± 5.65	60.13 ± 1.95	45.92 ± 5.21
	Benzaldehyde	—	26.61 ± 2.95	546.03 ± 38.37	306.03 ± 20.91	49.84 ± 5.46
Alcohols	2-Methyl-1-propanol	3.66 ± 0.65	31.31 ± 2.38	—	—	—
	2-Ethylhexanol	—	—	—	11.14 ± 1.04	46.19 ± 3.98
	2-Phenylethanol	1.87 ± 0.03	—	—	—	—
	1-Ethylcyclohexanol	—	21.54 ± 2.38	22.03 ± 1.37	18.35 ± 1.64	12.85 ± 0.22
Acids	Acetic acid glacial	1.72 ± 0.11	35.17 ± 2.51	137.85 ± 2.83	162.09 ± 10.44	17.72 ± 2.84
	Isobutyric acid	11.12 ± 1.93	51.87 ± 2.85	420.56 ± 10.12	369.54 ± 19.80	225.88 ± 18.58
	Isovaleric acid	28.55 ± 3.45	102.92 ± 9.13	1604.38 ± 104.13	1622.43 ± 115.29	1811.94 ± 130.24
	Butyric acid	—	—	59.59 ± 1.61	90.06 ± 1.35	—
	DL-2-methylbutyric acid	—	—	966.20 ± 8.33	—	—
	4-Methylvaleric acid	—	—	333.38 ± 6.87	105.22 ± 6.28	29.34 ± 3.32
	4-Methyl-hexanoic acid	—	—	501.05 ± 13.93	151.29 ± 2.51	40.17 ± 6.64
	2-Butenoic acid,2-methyl-	—	—	—	53.67 ± 7.09	15.09 ± 2.01
	3,3-Dimethylacrylic acid	—	—	—	—	9.54 ± 1.07
Esters	Isobutyl butyrate	—	36.03 ± 3.10	—	—	—
	Formic acid, octyl ester	7.62 ± 0.28	—	—	—	—
Pyrazines	2,5-Dimethyl pyrazine	—	—	17.29 ± 0.22	21.41 ± 3.09	95.54 ± 9.18
	2,6-Dimethylpyrazine	—	—	40.10 ± 3.76	50.42 ± 6.27	59.44 ± 9.34
	2,3,5-Trimethylpyrazine	—	—	16.49 ± 0.37	48.84 ± 5.34	85.17 ± 6.63
	TTMP	—	—	—	14.24 ± 3.16	10.45 ± 0.66
Alkanes	Decane	6.18 ± 2.25	45.2 ± 2.47	62.74 ± 4.25	85.96 ± 6.85	—
	3,7-Dimethyl-cyclohexene	—	36.92 ± 3.81	38.80 ± 5.60	30.97 ± 4.72	18.87 ± 0.51
	Phenylethylene	—	52.27 ± 9.24	54.50 ± 3.06	52.98 ± 7.63	32.6 ± 4.59
	Naphthalene	11.29 ± 0.43	95.51 ± 7.94	135.85 ± 4.69	140.03 ± 1.78	33.90 ± 1.23

Pyrazines are important flavor substances in Moutai-flavor liquor, and those were suggested on the main sauce-like aroma components in liquor (Duan et al., 2022). Dimethylpyrazine (2,5-dimethyl pyrazine and 2,6-dimethylpyrazine) and trimethylpyrazine (2,3,5-trimethylpyrazine) are formed by the Maillard reaction of reducing sugars and amino acids (Koehler et al., 1969; Amrani-Hemaimi et al., 1995). Some searches showed that dimethylpyrazine could by synthesized by *Bacillus subtilis*, and dimethylpyrazine might be related to produce trimethylpyrazine (Dickschat et al., 2010; Zhang L. J. et al., 2019). 2,5-Dimethylpyrazine has a strong roasted aroma, and 2,3,5-trimethylpyrazine has a strong aroma reminiscent of roasted peanuts or potatoes. Those pyrazines commonly found in baked goods, cocoa products, coffee, and other food items (Scalone et al., 2020). TTMP is a key characteristic aroma component of Moutai-flavor liquor and a bioactive alkaloid found in medicinal plants used to treat cardiovascular and cerebrovascular diseases (Jia et al., 2020; Lin et al., 2022). Some studies indicated that TTMP is generated by a condensation reaction of the acetoin and amino acid, and this chemical reaction is a thermodynamic reaction, which can produce biochemical reaction at 37°C, and the content increased with fermentation temperature of bacteria (Wu and Xu, 2014; Guo et al., 2022). Meanwhile, we detected a sauce-like aroma in the fermentation broth, indicating that pyrazines may be synthesized through microbial metabolism and thermodynamic reactions.

### 3.3 Quality check of transcriptome sequencing data

The original data were obtained by transcriptome sequencing of the samples at 24 and 96 h of fermentation. The quality control software FASTP was used to control the quality of the data to reduce the noise interference of the sample data, and the effective data were obtained. As shown in Table 2, the samples at 24 (labeled as C) and 96 h (labeled as A) of fermentation obtained 18,540,010–20,736,290 bp and 18,562,826–22,873,952 bp clean reads, respectively, and the high-quality read ratio was more than 99%. In addition, Q20, Q30, and GC percentages of the samples are more than 98, 95, and 52%, respectively. The reads after filtering ribosomes were aligned to the reference genome, and the results were shown in Table 3. The alignment rate of each sample with the reference genome was greater than 98%, indicating that the reference gene sequence was appropriate (Shi et al., 2023).

**Table 2:** The sequencing data quality of the samples fermented at 24 (labeled as C) and 48 h (labeled as A), respectively.

Samples	Raw reads	Clean reads (%)	Q20 (%)	Q30 (%)	GC (%)
A1	18,590,314	18,540,010 (99.73%)	98.47	95.42	52.68
A2	20,783,656	20,736,290 (99.77%)	98.57	95.78	52.44
A3	20,006,172	19,959,052 (99.76%)	98.57	95.52	52.30
C1	19,266,582	19,223,746 (99.78%)	98.57	95.58	52.58
C2	18,602,586	18,562,826 (99.79%)	98.57	95.72	52.49
C3	22,922,740	22,873,952 (99.79%)	98.57	95.42	52.60

**Table 3:** Statistical sequence results of the reference genome of the samples fermented at 24 (labeled as C) and 48 h (labeled as A), respectively.

Samples	Total reads	Unmapped reads	Unique mapped reads	Multiple mapped reads	Mapping ratio (%)
A1	18,394,016	117,179 (0.64%)	17,566,637 (95.50%)	710,200 (3.86%)	99.36
A2	20,541,294	198,580 (0.97%)	19,387,542 (94.38%)	955,172 (4.65%)	99.03
A3	19,786,480	198,052 (1.00%)	18,770,972 (94.87%)	817,456 (4.13%)	99.00
C1	18,898,802	239,924 (1.27%)	16,856,707 (89.19%)	1,802,171 (9.54%)	98.73
C2	18,284,518	181,860 (0.99%)	16,415,408 (89.78%)	1,687,250 (9.23%)	99.01
C3	22,575,272	151,913 (0.67%)	20,222,544 (89.58%)	2,200,815 (9.75%)	99.33

### 3.4 Results of DEGs and bioinformatics analysis

**Figure 3:**
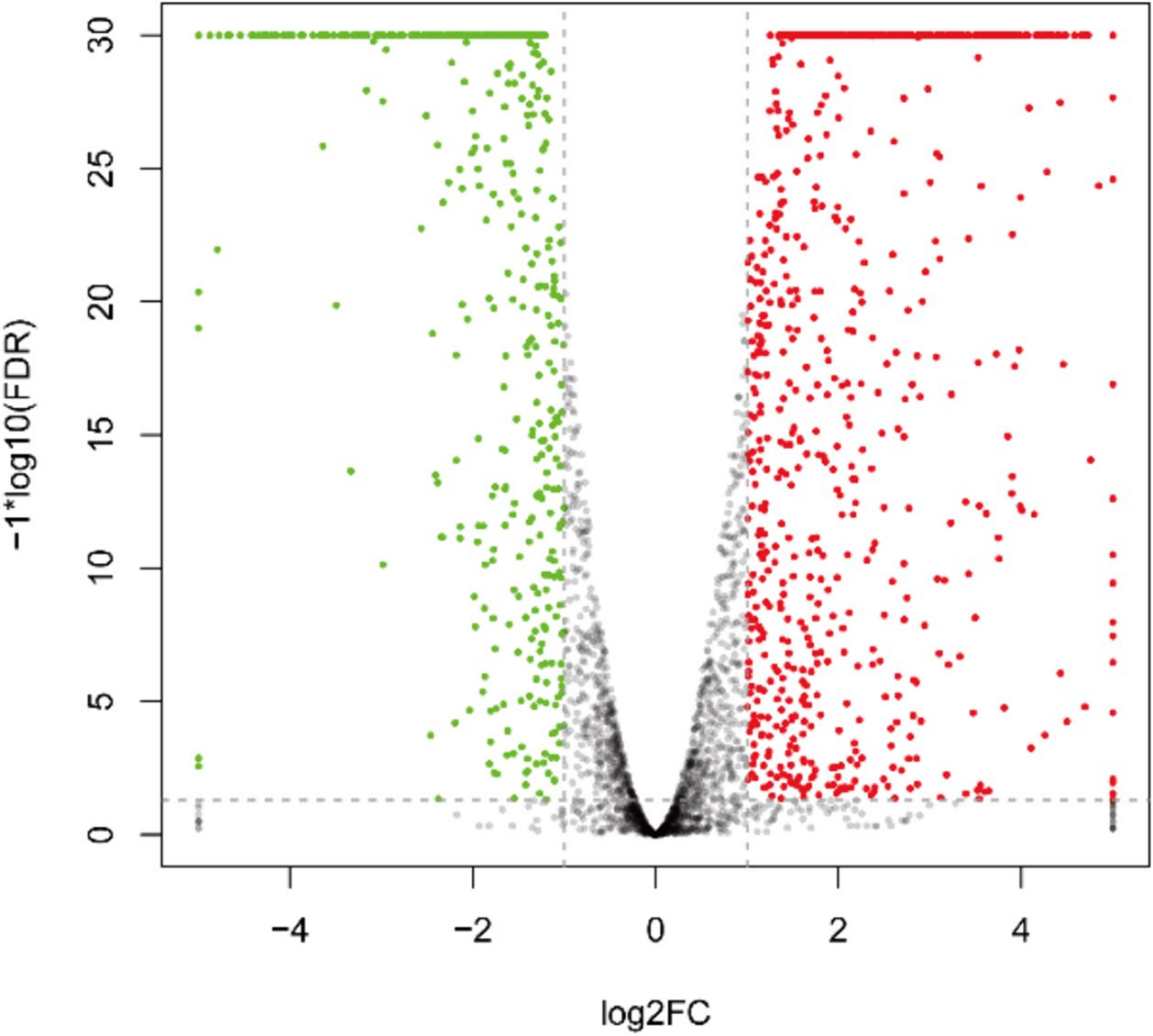
Inter-group differential gene volcano plot of DEGs. The green pots indicate downregulation, red pots indicate upregulation, and black pots indicate no significant difference.

As depicted in Figure 3 and Supplementary Table S1, the transcriptome sequencing yielded a total of 1,284 DEGs, with 808 up-regulated and 476 down-regulated genes.

**Figure 4:**
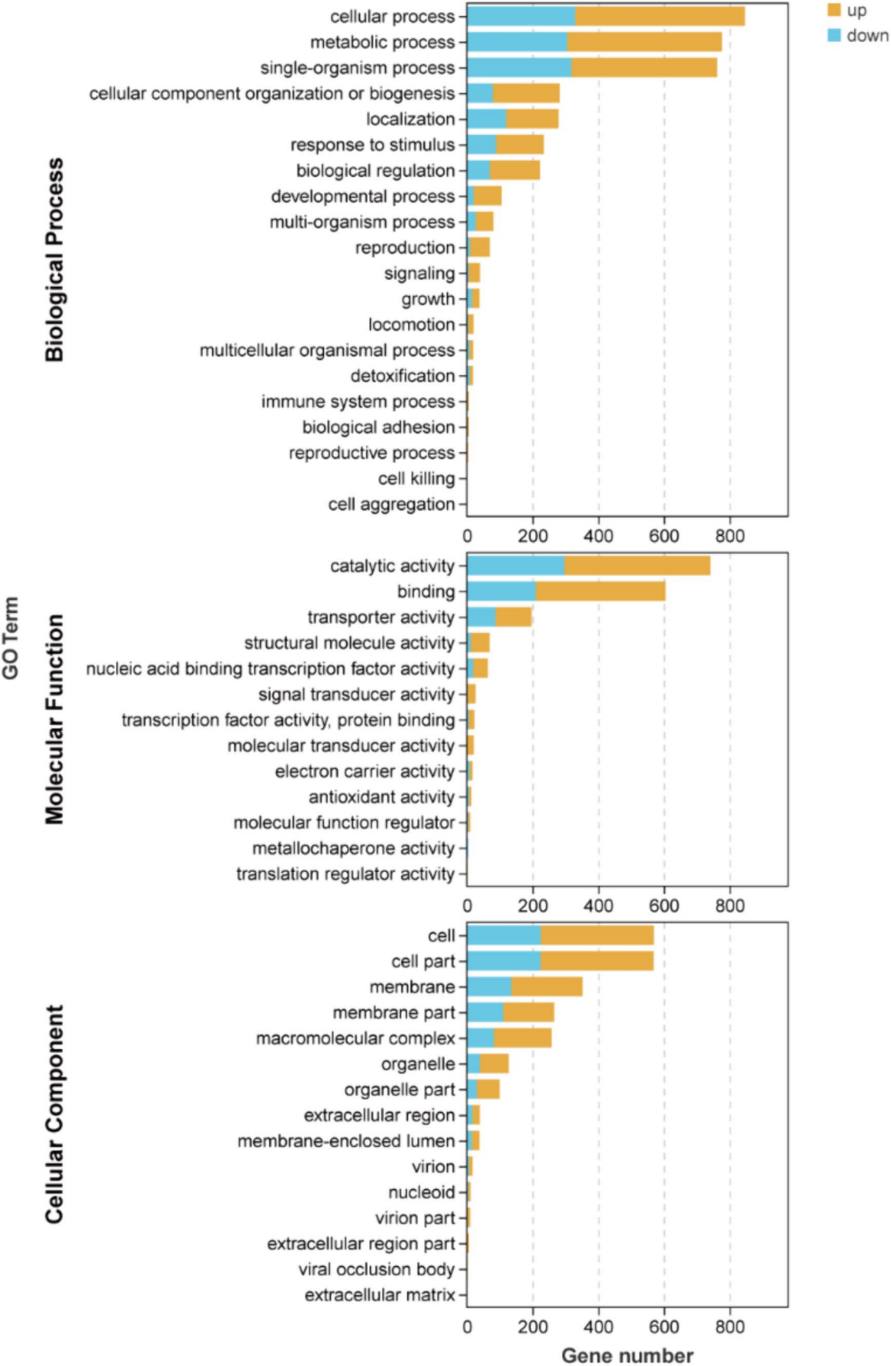
Inter-group differential gene GO classification bar chart of DEGs.

The results depicted in Figure 4 and Supplementary Table S2 demonstrate that there are 19, 16, and 13 terms associated with BP, CC, and MF, respectively. Among these terms in BP, the metabolic process (471 up-regulated and 306 down-regulated), cellular process (518 up-regulated and 329 down-regulated), and single-organism process (444 up-regulated and 318 down-regulated) exhibited the highest enrichment of genes. Similarly, in MF, catalytic activity (444 up-regulated and 297 down-regulated), binding (395 up-regulated and 210 down-regulated), and transporter activity (110 up-regulated and 87 down-regulated) were identified as the top three terms with significant gene enrichment. Furthermore, within CC category, cell (345 up-regulated and 225 down-regulated), cell part (345 up-regulated and 224 down-regulated), and membrane (217 up-regulated and 135 down-regulated) were found to be the three terms with the most pronounced gene enrichment. It’s showed that DEGs about cellular process and metabolic of BP, catalytic activity and binding of MF, and cell, cell part, and membrane of CC were significantly upward and downward revisions after 96 h in the fermentation process.

**Figure 5:**
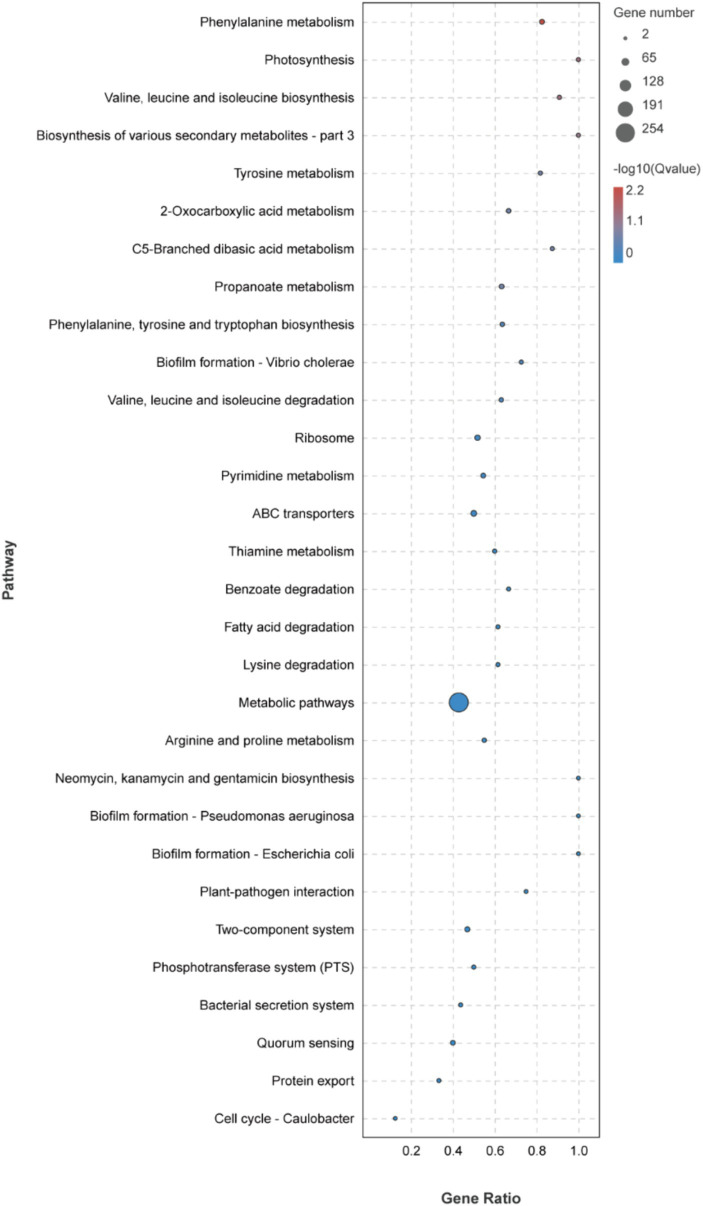
Top 30 KEGG metabolic pathways enrichment of DEGs.

The KEGG metabolic pathway enrichment analysis was conducted for the DEGs, resulting in the identification of 116 metabolic pathways (Supplementary Table S3). To facilitate visualization and comprehension, as depicted in Figure 5, we selected the top 30 metabolic pathways for presentation. Meanwhile, Table 4 demonstrates that the metabolic pathways exhibited the highest gene count, accounting for 90.91% in 9 significantly differentially enriched pathways. According to metabolic pathways, the gene expression of succinyl-CoA synthesis from phenylalanine metabolism and propanoate metabolism, tyrosine and acetoacetate synthesis from tyrosine metabolism, propanoate synthesis from propanoate metabolism, prephenate, phenyl-pyruvate, phenyl-alanine and tyrosine synthesis from phenylalanine, tyrosine and tryptophan biosynthesis pathway were down-regulated. Then, the gene expression of L-isoleucine, L-valine, and L-leucine synthesis from valine, leucine, and isoleucine biosynthesis pathway and valine, leucine, and isoleucine degradation pathway, 3-methoxy-4-hydroxy-mandelate synthesis from tyrosine metabolism, omithine, valine, leucine, and isoleucine synthesis from 2-oxocarboxylic acid metabolism, (*R*)-citramalate, 2-oxobutanoate, and (*R*)-acetoin synthesis from C5-branched dibasic acid metabolism, L-tryptophan and *N*-(5-phospho-β-D-hbosyl)-anthranilate synthesis from phenylalanine, tyrosine and tryptophan biosynthesis pathway were up-regulated. Meanwhile, we found that gene expression of valine, leucine, isoleucine, serine, methionine, cysteine, homoserine and citrulline synthesis from biosynthesis of amino acids pathway was up-regulated. In addition, the serine degradation pathway gene expression from biosynthesis of various secondary metabolites-part 3, oxaloacetate degradation pathway gene expression from 2-oxocarboxylic acid metabolism, L-isoleucine, L-valine, and L-leucine synthesis from valine, leucine, and isoleucine degradation pathway were down-regulated.

**Table 4:** Top nine significantly different KEGG metabolic pathways.

Pathway ID	Pathway name	*p*-value
ko00360	Phenylalanine metabolism	0.0000526
ko00290	Valine, leucine, and isoleucine biosynthesis	0.000954111
ko00997	Biosynthesis of various secondary metabolites	0.002015161
ko00350	Tyrosine metabolism	0.007447992
ko00660	C5-Branched dibasic acid metabolism	0.01034393
ko00640	Propanoate metabolism	0.01148237
ko01210	2-Oxocarboxylic acid metabolism	0.01016241
ko00400	Phenylalanine, tyrosine, and tryptophan biosynthesis	0.02798384
ko00280	Valine, leucine, and isoleucine degradation	0.04456314

**Figure 6:**
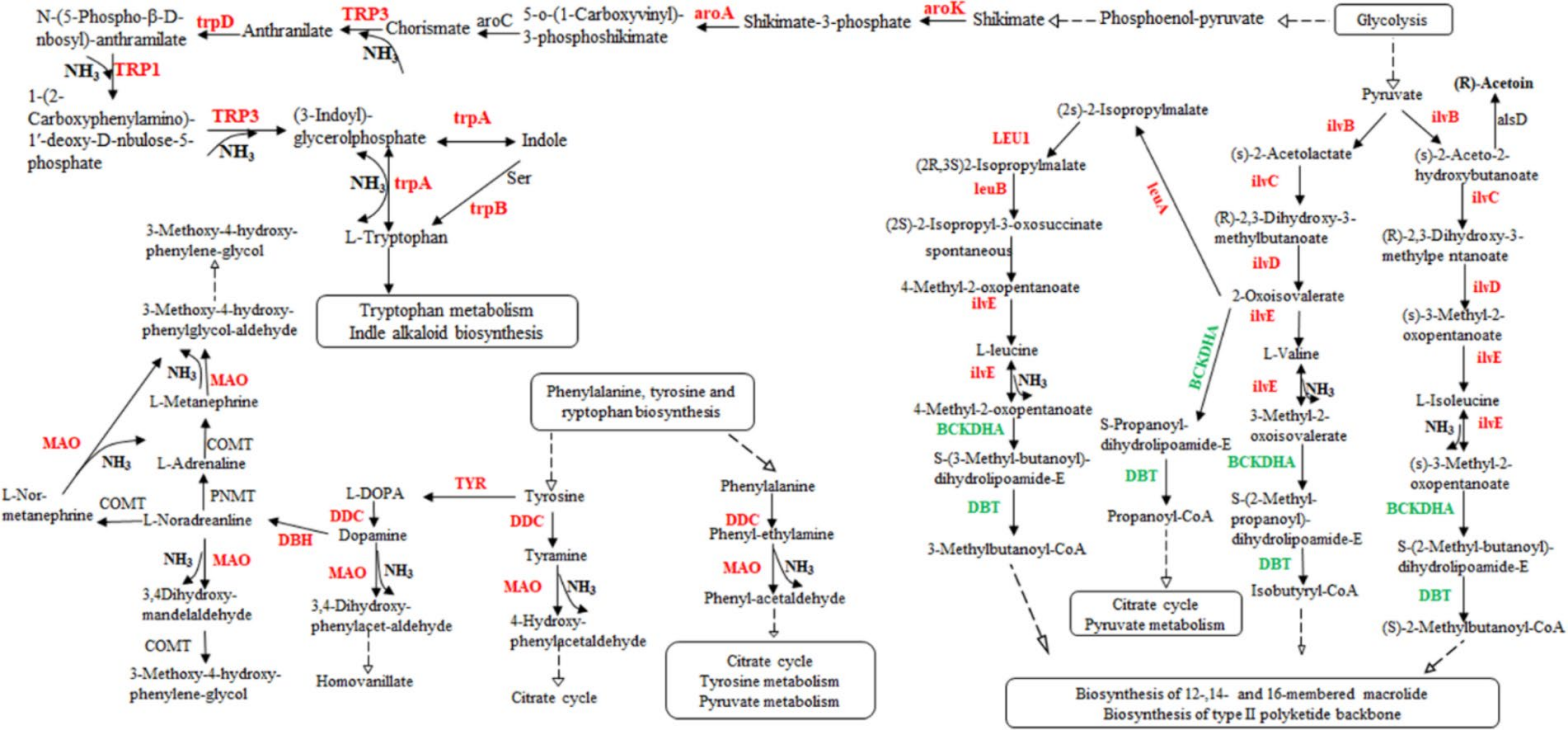
The valine, leucine, and isoleucine biosynthesis pathway in *L. sacchari*. Red: up-regulated genes; Green: down-regulated genes. DDC, L-tryptophan decarboxylase; MAO, monoamine oxidase; ilvB, acetolactate synthase; ilvC, ketol-acid reductoisomerase; ilvD, dihydroxy-acid dehydratase; ilvE, branched-chain amino acid aminotransferase; leuA, 2-isopropylmalate synthase; LEU1, 3-isopropylmalate dehydratase; leuB, 3-isopropylmalate dehydrogenase; BCKDHA, 2-oxoisovalerate dehydrogenase; DBT, dihydrolipoyl transacylase; TYR, tyrosinase; DBH, dopamine beta-monooxygenase; PNMT, phenylethanolamine N-methyltransferase; COMT, catechol O-methyltransferase; alsD, acetolactate decarboxylase; aroK, shikimate kinase; aroA, 3-phosphoshikimate 1-carboxyvinyltransferase; TRP3, anthranilate synthase/indole-3-glycerol phosphate synthase; aroC, chorismate synthase; trpD, anthranilate phosphoribosyltransferase; TRP1, anthranilate synthase; TRP3, anthranilate synthase; trpA, tryptophan synthase alpha chain.

According to the transcriptome sequencing data, we observed an upregulation in gene expression related to acetoin synthesis, accompanied by a gradual decrease in acetoin content during fermentation. However, there was an increase in pyrazine content, suggesting that acetoin might be utilized for pyrazine generation. Additionally, DEGs involved in isoleucine, valine, and leucine biosynthesis were upregulated, while those associated with isoleucine, valine, and leucine degradation were downregulated (Supplementary Table S4). These processes could potentially lead to the production of a significant amount of ammonia synthesizing TTMP using acetoin as a precursor. Although rRNA genes, the gene of threonine 3-dehydrogenase catalyzed threonine to L-2-amino acetoacetate, were not found in the transcriptome data, 2,5-DMP and TMP were generated by enzymes. Those genes of enzymes might not be annotated to the strain genome. Moreover, some results showed that *Bacillus subtilis* could produce 2,5-DMP and TMP by threonine dehydrogenase, and Maillard reaction could produce at least 10 kinds of pyrazines (Van Lancker et al., 2010; Hu et al., 2024). Therefore, the products, 2,5-DMP, 2,6-DMP, TMP, and TTMP, might be generated by combining with enzyme and thermodynamic reaction in the fermentation of *L. sacchari*. These findings indicate that the biosynthesis of isoleucine, valine and leucine was primarily contribute ammonia for TTMP synthesis (Figure 6).

## 4 Conclusion

In this study, we investigated the liquid fermentation characteristics of *L. sacchari*. Furthermore, through analysis of volatile components and comparative transcriptomic techniques, it was determined that the valine, leucine, and isoleucine biosynthesis pathway primarily contributes ammonia for TTMP synthesis. Therefore, our findings are significant in terms of enhancing the fermentation quality and flavor characteristics of Chinese liquor.

## Data availability statement

The original contributions presented in the study are included in the article/Supplementary material, further inquiries can be directed to the corresponding authors.

## Author contributions

XW: Funding acquisition, Formal analysis, Data curation, Conceptualization, Writing – review & editing, Writing – original draft, Methodology. WH: Investigation, Writing – original draft, Formal analysis, Conceptualization. JH: Methodology, Data curation, Writing – original draft. XL: Investigation, Writing – original draft. MN: Writing – original draft, Investigation. TJ: Writing – original draft, Investigation. SB: Writing – review & editing, Validation, Supervision, Resources, Project administration, Methodology, Data curation, Conceptualization, Writing – original draft. PL: Writing – review & editing, Writing – original draft, Methodology.

## Footnotes

^1^ http://www.r-project.org/

## Supplementary material

Supplementary TABLE S1 All DEGs detected by the transcriptome sequencing.

Supplementary TABLE S2 GO enrichment analysis of DEGs.

Supplementary TABLE S3 KEGG metabolic pathways enrichment of DEGs.

Supplementary TABLE S4 DEGs involved in isoleucine, valine, and leucine biosynthesis and isoleucine, valine, and leucine degradation.
